# Chronic active Epstein–Barr virus infection manifesting as coronary artery aneurysm and uveitis

**DOI:** 10.1186/s12985-020-01409-8

**Published:** 2020-10-29

**Authors:** Haijuan Xiao, Bing Hu, Rongmu Luo, Huili Hu, Junmei Zhang, Weiying Kuang, Rui Zhang, Li Li, Gang Liu

**Affiliations:** 1 Department of Infectious Diseases, Beijing Children’s Hospital, Capital Medical University, National Center for Children’s Health, Beijing, China; 2 Department of Hematology and Oncology, Affiliated Bayi Children’s Hospital, The Seventh Medical Center of PLA General Hospital, Beijing, China; 3 Department of Rheumatology, Beijing Children’s Hospital, Capital Medical University, National Center for Children’s Health, Beijing, China; 4 Hematology Oncology Center, Beijing Children’s Hospital, Capital Medical University, National Center for Children’s Health, Beijing, China; 5 Department of Ophthalmology, Beijing Children’s Hospital, Capital Medical University, National Center for Children’s Health, Beijing, China

**Keywords:** Chronic active Epstein–Barr virus infection (CAEBV), Coronary artery aneurysm (CAA), Coronary artery ectasia (CAE), Lymphoproliferative disorders (LPDs), Uveitis, Hematopoietic stem cell transplantation (HSCT)

## Abstract

**Background:**

Chronic active Epstein–Barr virus (CAEBV) infection is a type of lymphoproliferative disorder characterized by chronic or recurrent infectious mononucleosis (IM)-like symptoms, which can have less-frequent clinical presentations. The prognosis of CAEBV is poor, and hematopoietic stem cell transplantation (HSCT) has been shown to be the only potentially effective treatment. In this article, we present a special CAEBV case of a patient who had no typical IM-like symptoms at the early stage, but manifested with severe and progressive coronary artery aneurysm (CAA), abdominal aortic lesions, and severe uveitis. These manifestations were uncommon features and could only be blocked by HSCT.

**Case presentation:**

A 4-year-old girl with no special medical history complained of decreased vision for 10 months and cough after physical activities for three months. The blurred vision grew rapidly worse within one month, until only light perception remained. She was diagnosed with uveitis and cataract, and received prednisone and ciclosporin A treatment. However, her vision did not improve. Physical examination showed slight hepatosplenomegaly. Ultrasonic cardiogram showed bilateral CAA (5.0 mm and 5.7 mm for inner diameters), and abdominal CT scan revealed a thickened aortic wall, as well as stenosis and dilation of the segmental abdominal aorta. Other significant findings were increased EBV-DNA (3.29 × 10^4^ copies/mL) from peripheral blood, positive EBV antibodies (EBV-CA-IgG, EBV-EA-IgA, and EBV-NA-IgG), and positive EBV-encoded small RNAs found by bone marrow biopsy. Based on her clinical manifestations and evidence for EBV infection, we diagnosed CAEBV. She received allogeneic HSCT, and the cataract operation was performed after HSCT. EBV-DNA could not be detected in peripheral blood after HSCT. Her CAAs did not progress, and uveitis was well controlled. Her vision recovered gradually over the 3 years after HSCT.

**Conclusions:**

We present a rare CAEBV case of a patient who suffered from uncommon and severe cardiovascular and ocular involvement that was relieved by HSCT. Therefore, early recognition and diagnosis of CAEBV are of vital importance to improve its prognosis. In summary, this atypical CAEBV case could help us recognize similar cases more easily, make the right diagnosis as early as possible, and deliver proper and timely treatment.

## Background

Epstein–Barr virus (EBV) is a ubiquitous virus infecting more than 90% of the population worldwide. EBV infection in humans is usually asymptomatic and persists as a lifelong latent infection [1]. However, the infection or reactivation of EBV could result in various lymphoproliferative disorders (LPDs), including infectious mononucleosis (IM) and hematologic malignancies [2]. Chronic active EBV infection (CAEBV) is a severe disease with high morbidity and mortality, which exhibits a predisposition in East Asian populations. As a type of LPD, the clonal expansion of EBV-infected T or NK cells plays a central role in the disease’s pathogenesis. However, the detailed pathogenesis of CAEBV and the mechanism by which EBV induces proliferation of T and NK cells are not known [1]. CAEBV is characterized by chronic or recurrent IM-like symptoms, such as fever, hepatosplenomegaly, lymphadenopathy, and liver dysfunction. However, CAEBV can also have other less frequent clinical presentations, or even fetal complications, such as central nervous system involvement, coronary artery aneurysm (CAA), interstitial pneumonia, digestive tract disorders, uveitis, and hemophagocytic lymphohistiocytosis (HLH) [3]. The prognosis of CAEBV is poor, and a series of therapies has been attempted, including anti-viral agents and immunosuppressors. However, hematopoietic stem cell transplantation (HSCT) has been shown to be the only potentially effective treatment [1].

Clonal proliferation of EBV-infected T or NK cells implies that CAEBV has a malignant nature. However, CAEBV is a chronic disease, and patients may remain in a stable condition for years without effective treatment [4]. Overt malignant lymphoma often occurs after a long course of disease. Some researchers propose that CAEBV is a continuous spectrum ranging from a smoldering phase to overt lymphoma [4]. It is commonly believed that hosts with normal immune functions possess the ability to recognize EBV-infected T and NK cells, and CAEBV patients are thus thought to have some defects in immunological function that cause inefficient recognition and/or killing of EBV-infected cells [1]. However, CAEBV patients have not been found to have obvious immunodeficiency until the present day [5]. Nevertheless, it should be noted that some immunocompromised patients have accompanying EBV infections, and their clinical manifestations are analogous to CAEBV [6]. The mutations of several genes, including *SH2D1A, XIAP, CD27, CD70, MAGT1,* and *PRKCD,* are shown to cause hosts to be susceptible to chronic or even fetal EBV infections [7]. Therefore, genetic testing is necessary to distinguish CAEBV from these primary immunodeficiency diseases (PIDs).

Coronary artery ectasia (CAE) is an uncommon cardiovascular disorder that is defined as localized or diffuse dilatation of the coronary lumen. CAA describes local dilatation in the coronary lumen that is 1.5-fold greater than in normal adjacent segments [8]. CAA could be seen in various disorders, including atherosclerosis, systemic inflammatory vasculitis (e.g., Kawasaki disease, Behcet’s disease), hereditary collagen defects (e.g., Marfan syndrome), infectious diseases (e.g., bacteria, mycobacteria), and congenital malformations [9]. Uveitis describes inflammation of the uvea, which contains the iris, ciliary body, and choroid. As intraocular inflammation can affect surrounding tissues, clinical uveitis may comprise inflammation of the retina, optic disc, and vitreous [10]. Uveitis may be the result of infectious (e.g., human herpes virus, tuberculosis, syphilis), non-infectious (mostly autoimmune or autoinflammatory), or masquerade (e.g., lymphoma) causes [11]. In this article, we will introduce a special CAEBV case of a patient who had no typical IM-like symptoms at the early stage, but whose illness manifested as uveitis, cataract, and cardiovascular involvement (CAA).

## Case presentation

In July 2016, a 4-year-old girl was admitted to our department complaining of decreased vision and cough after physical activities. Ten months before hospitalization, she suffered from blurred vision, which grew rapidly worse within one month, until only light perception remained. In her local hospital, she was diagnosed with uveitis and cataract, and received prednisone, ciclosporin A, and local symptomatic treatment. The ocular lesions did not further exacerbate. Three months before hospitalization, the girl began to cough after exercise, with no fevers or other symptoms. Examinations showed normal blood routines and biochemical indicators, as well as a slightly increased erythrocyte sedimentation rate (ESR, 29 mm/h). There were no positive findings from the pulmonary CT scan or electrocardiogram, while an ultrasonic cardiogram showed bilateral CAAs, hypertrophic interventricular septum and left ventricular wall, and mitral and aortic valve insufficiency. Other significant findings were increased EBV-DNA (3.29 × 10^4^ copies/mL) in the peripheral blood, and positive EBV antibodies (EBV-CA-IgG, EBV-EA-IgA, and EBV-NA-IgG). She was given aspirin and ganciclovir, and the cough subsided. This patient did not suffer from recurrent infections, having no other medical history and no family history of PID. She had an elder sister (17 years old), who was healthy with no known diseases. Physical examination showed vision loss (only light perception remaining), systolic murmur at the apex, and slight hepatosplenomegaly.

**Fig. 1:**
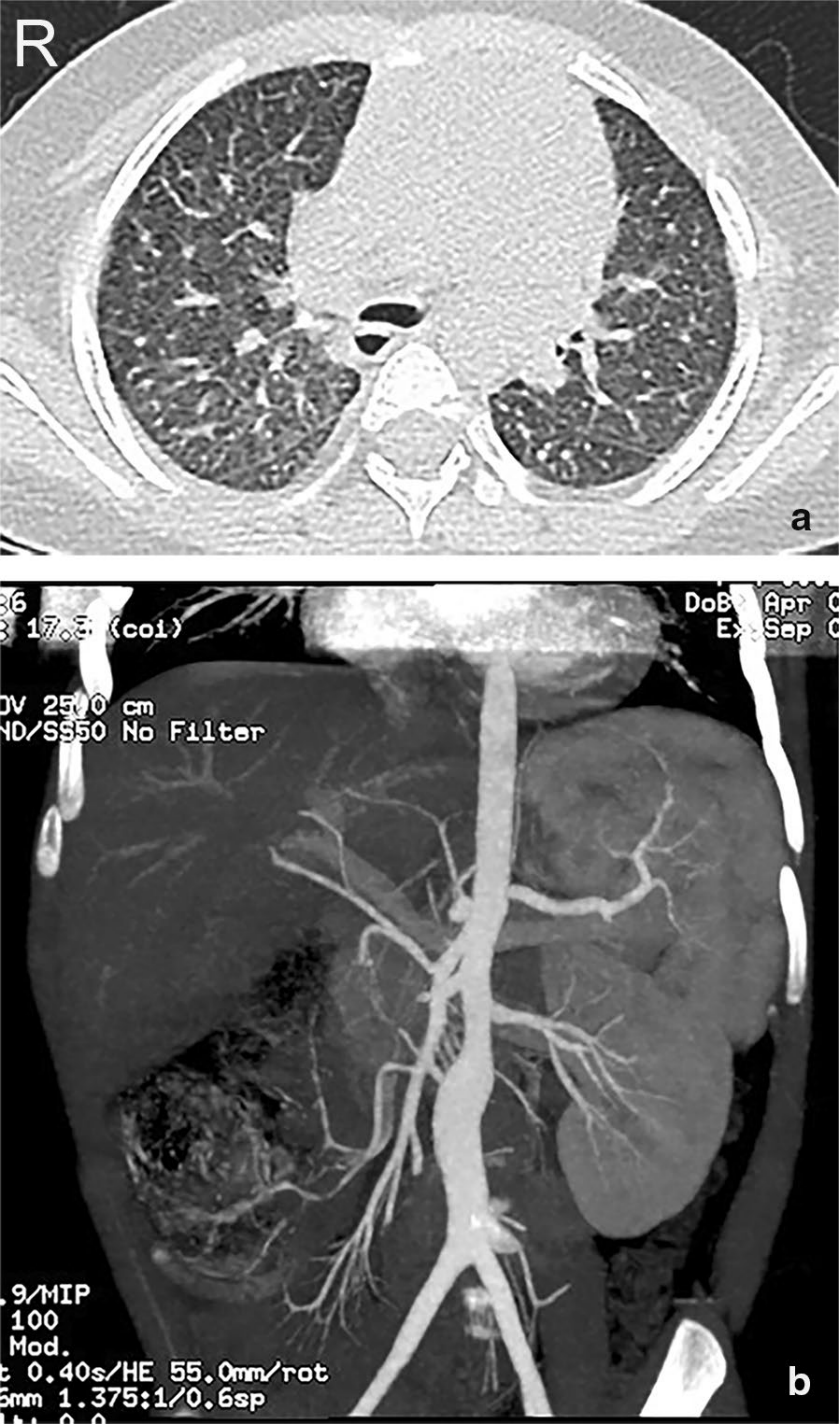
Chest and abdominal CT scan findings. **a** Extensively parenchymal and interstitial lesions of bilateral lungs are shown. **b** Thickened aortic wall, and stenosis and dilation of segmental abdominal aorta are shown

**Fig. 2:**
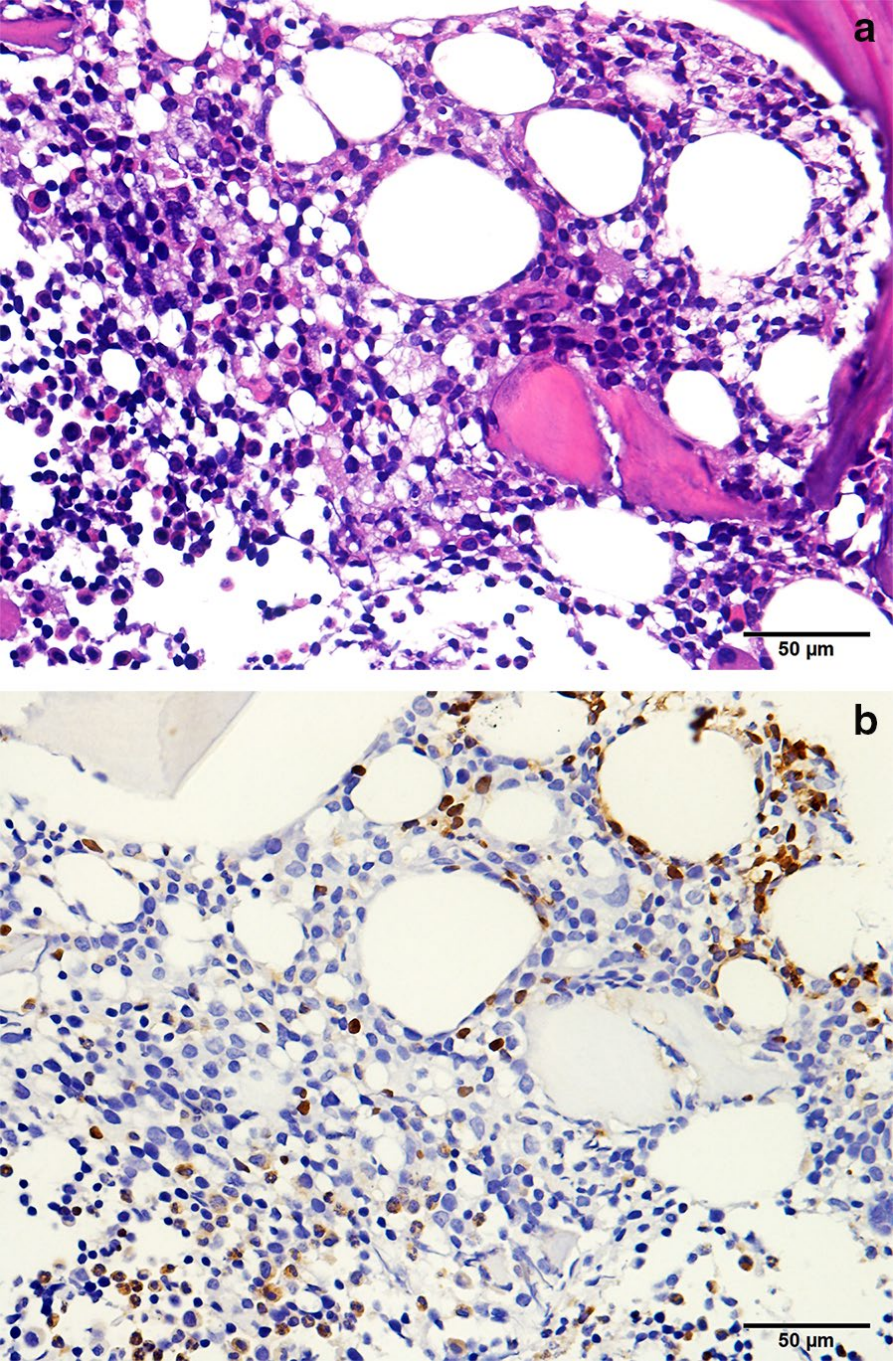
Pathological results for bone marrow biopsy. **a** Hematoxylin and eosin staining show much infiltration of lymphoid cells with mildly irregular nuclei. **b** In-situ hybridization for EBERs is positive

The examinations after hospitalization showed generally normal brain MRI manifestations, and similar results for ophthalmic tests and the ultrasonic cardiogram as before (inner diameters of left and right coronary arteries: 5.0 mm and 5.7 mm respectively). The pulmonary CT scan showed extensive parenchymal and interstitial lesions of the lungs bilaterally (Fig. 1a), and abdominal CT scan revealed a thickened aortic wall, as well as stenosis and dilation of the segmental abdominal aorta (Fig. 1b). There were no apparent abnormal findings in other vessels. Blood EBV-DNA and EBV antibodies were still positive, but autoantibodies were negative. There was no evidence for other infections, including other human herpes viruses [such as herpes simplex virus (HSV), varicella zoster virus (VZV), cytomegalovirus (CMV), and human herpes virus 8 (HHV-8)], human immunodeficiency virus (HIV), tuberculosis, toxoplasmosis, and syphilis. The immunoglobulin and complement levels were within normal range. Lymphocyte subgroups showed an increased percentage of CD3+ T cells and a decreased percentage of B cells and NK cells. The proportion of CD4+ T cells increased, and the ratio of CD4+ T to CD8+ T cells was also elevated (Table 1). A bone marrow smear was near normal, while bone marrow biopsy showed much infiltration of lymphoid cells, which had mildly irregular nuclei (Fig. 2a). Positive CD3/CD5/CD7/CD2 (partially)/TIA-1/GrB (sparsely)/ki67 (80%) and negative CD20/CD56 were revealed by immunohistochemistry, and the presence of EBV-encoded small RNAs (EBERs) was shown by in-situ hybridization (Fig. 2b). We did not find significant pathogenic genes by whole-exome sequencing (WES).

**Table 1:** Lymphocyte subsets before and 1 year after HSCT

	Before HSCT	1 year after HSCT	Reference values^a^
CD3+ T lymphocytes (%)	91.4	85.7	55–82
CD3+CD4+ T lymphocytes (%)	74.5	36.0	27–57
CD3+CD8+ T lymphocytes (%)	15.4	41.0	14–33
CD4/CD8	4.8	0.9^b^	1.1–2
CD19+ B lymphocytes (%)	5.1	2.1	9–29
CD16+CD56+ NK cells (%)	1.5	10.2	7–40

^a^Reference values used in Beijing Children’s Hospital

^b^The value of another test at the same time was 1.25

Based on her clinical manifestations, increased EBV-DNA, positive EBV antibodies, and the pathological results of the bone marrow biopsy, we diagnosed the patient with CAEBV. She received allogeneic HSCT (allo-HSCT) in another hospital. The EBV-DNA in the peripheral blood could not be detected after HSCT. Compared with before HSCT, the percentage of CD4+ T cells and the ratio of CD4+ T to CD8+ T cells both decreased after HSCT (Table 1). The cataract operation was performed after HSCT. Her CAAs did not progress, and uveitis was well controlled. The patient’s vision recovered gradually over the 3 years after HSCT.

## Discussion and conclusions

This is a rare CAEBV case of a patient who suffered from cardiovascular and ocular involvement. This patient showed severe and progressive CAAs and abdominal aortic lesions, which could only be blocked by HSCT. Uveitis was also controlled, and vision recovered gradually after HSCT. She did not have typical IM-like symptoms at the early stage, and slight hepatosplenomegaly did not occur until the very late stage. Therefore, early recognition and diagnosis of CAEBV are of vital importance to improve the prognosis of patients like the case described.

There have been several reports about CAEBV-associated cardiovascular diseases, including CAA, aortic aneurysms, myocarditis, pericardial effusion, and others, among which CAA and myocarditis are the major complications resulting in a poor prognosis [12, 13]. In Table 2, we summarize 20 CAEBV patients with coronary artery lesions reported in the literature, including 11 females and 9 males. There were 19 cases who had detailed descriptions about clinical features. The age at onset of CAEBV was between 2 years old and 16 years old, and 16 patients had symptoms at less than 10 years old. Eighteen patients had IM-like symptoms during the disease course. CAE, or even CAA, was found nearly simultaneously with CAEBV diagnosis in 12 cases, while these were found about 2–9 years after diagnosis in seven patients. Coronary lesions were described in detail in 13 patients: there were eight cases with bilateral CAE, four cases with left CAE, and one with right CAE only. The most severe coronary diameter was 8.2 mm. Only seven cases suffered from concomitant vascular lesions in the major branches of aorta. Three patients with pericardial effusion and two patients with pulmonary arterial hypertension (PAH) were noted, but no myocarditis was reported. Other organ lesions included pneumonia, nephritis, gastrointestinal diseases, and skin lesions. EBV-infected cell types were revealed in 12 cases: including 10 patients with infected T cells, one with infected NK cells, and one with infected γδT cells. Seventeen patients showed evidence for EBV infections, and all of them had positive EBV-DNA or EBERs. However, it should be noted that EBV antibodies were negative in one patient. As for the therapy, HSCT were not performed in 12 cases, among which eight patients died for various reasons. Six patients experienced HSCT: two patients were alive, and three patients died.

**Table 2:** Clinical manifestations and prognosis of previously reported CAEBV patients with coronary artery lesions

Pt	Sex	Onset age (years)	IM-like symptoms	Coronary artery lesions			Other cardiovascular complications			Other organ manifestations
				Occurrence age (years)	Affected branch	Coronary diameter	Large-vessel arteritis	Myocarditis	Others	
1	M	4	L, H	4	NA	NA	No	No	No	Interstitial pneumonitis, Sjögren syndrome
2	F	5	L, H	5	NA	NA	Abdominal aortic aneurysm	No	No	Interstitial pneumonitis
3	M	2	F, L, H	2	NA	NA	Dilation of the sinus of Valsalva	No	No	Interstitial pneumonitis
4	F	6	F, L, H	6	NA	NA	No	No	Pericarditis	Interstitial nephritis
5	F	5	F, H	5	NA	NA	Dilation of the sinus of Valsalva	No	Pericarditis	No
6	F	2	F, H	4	LCA	4 mm	No	No	No	Gastrointestinal tract, hypersensitivity to mosquito bites, hydroa vacciniforme
7	M	5	F, H	8	LCA	5 mm	No	No	No	No
8	M	6	F, H	10	LCA	4.5 mm	No	No	No	No
9	F	9	F, H	18	LCA, RCA	4 mm in RCA, 4 mm in LCA	No	No	No	No
10	M	11	H	14	LCA, RCA	NA	No	No	PAH, junctional ectopic tachycardia	No
11	F	10	F, H	10	LCA, RCA	NA	Dilation of bilateral common carotid and subclavian arteries, abdominal aorta and major branches, and bilateral common iliac arteries	No	No	No
12	M	2	F, H, L	2	LCA, RCA	6 mm in RCA, 3 mm in LCA	No	No	No	No
13	F	7	F, H, L	7	NA	NA	No	No	Pericardial effusion	No
14	M	2	F, H, L	2	LAD, LCX	6 mm in LMCA	No	No	No	No
15	F	5	F, H	5	LCA, RCA	8 mm in RCA, 5 mm in LCA	38 mm in the sinus of Valsalva, thoracic and abdominal aortic aneurysms	No	No	No
16	M	6	F, H	8	NA	NA	No	No	No	No
17	F	NA	NA	NA	LCA, RCA	NA	No	No	No	No
18	F	16	F, H, L	16	RCA (multiple stenoses and dilation)	NA	Aneurysms involving the aorta and its major branches, and bilateral common iliac arteries	No	No	No
19	F	6	F, H, L	6	LMCA, RCA	5 mm in LMCA, 8.2 mm in RCA	No	No	No	No
20	M	5	NA	9	LAD, RCA	NA	Dilatation of aortic sinus, minor dilatation of abdominal aortic stem and the distal section of superior mesenteric artery	No	PAH, cardiac insufficiency	Skin ulcers

Pt	Sex	Onset age (years)	IM-like symptoms	EBV status			Therapies—HSCT	Prognosis	Reports
				Cell type	EBV copies (in PB)/EBERs	Abnormal EBV antibodies			
1	M	4	L, H	T	EBERs (+)	Yes	No	Died (streptococcus pneumoniae) within 36 months from onset	Nakagawa et al. [25]
2	F	5	L, H	T	EBERs (+)	Yes	No	Died (respiratory failure) within 13 months from onset	Nakagawa et al. [25]
3	M	2	F, L, H	NA	EBV-DNA in cardiac tissues	Yes	No	Died within 5 years from onset	Kikuta et al. [26]
4	F	6	F, L, H	NA	EBV-DNA in cardiac and aortic tissues	Yes	No	Died within 5 years from onset	Kikuta et al. [26]
5	F	5	F, H	NA	EBV-DNA in cardiac tissues	Yes	No	Died within 5 years from onset	Kikuta et al. [26]
6	F	2	F, H	NK	3 × 10^3^ copies/mL	No	Yes	Alive and disease free	Muneuchi et al. [12]
7	M	5	F, H	T	3 × 10^3^ copies/mL	Yes	Yes	Died at 14 years	Muneuchi et al. [12]
8	M	6	F, H	T	5 × 10^4^ copies/mL	Yes	Yes	Alive and disease free	Muneuchi et al. [12]
9	F	9	F, H	γδT	9 × 10^2^ copies/mL	Yes	No	Died at 18 years	Muneuchi et al. [12]
10	M	11	H	NA	8.2 × 10^4^ copies/μg DNA	Yes	Yes	Died (septic shock and multiple organ failure) within 13 days after transplantation	Fukuda et al. [27]
11	F	10	F, H	T	EBERs (+)	Yes	No	Died (respiratory failure due to diffuse alveolar damage)	Murakami et al. [28]
12	M	2	F, H, L	T	EBV-DNA in PBMC	Yes	NA	NA	Kikuta et al. [29]
13	F	7	F, H, L	T	EBV-DNA in pericardial effusion	Yes	Yes	Died (failure of umbilical cord blood transplantation)	Toubo et al. [30]
14	M	2	F, H, L	T	NA	Yes	No	NA	Kobayashi et al. [31]
15	F	5	F, H	T	EBERs (+)	Yes	No	Died (acute respiratory failure) within 13 months from onset	Nakagawa et al. [14]
16	M	6	F, H	T	NA	Yes	NA	NA	Ohga et al. [32]
17	F	NA	NA	NA	NA	NA	Yes	NA	Nishimura et al. [33]
18	F	16	F, H, L	NA	2.8 × 10^4^ copies/mL	NA	No	NA	Jiang et al. [34]
19	F	6	F, H, L	NA	3.72 × 10^7^ copies/mL	NA	No	NA	Xie et al. [35]
20	M	5	NA	NA	4.53 × 10^3^ copies/mL in plasma, EBERs (+)	Yes	No	NA	Ba et al. [36]

*NA* not available, *F* fever, *H* hepatosplenomegaly, *L* lymphadenopathy, *LAD* left anterior descending artery, *LCA* left coronary artery, *LCX* left circumflex artery, *LMCA* left main coronary artery, *RCA* right coronary artery, *PAH* pulmonary arterial hypertension, *PB* peripheral blood, *PBMC* peripheral blood mononuclear cell

The potential mechanism of CAA in CAEBV patients has not yet been discovered. The pathological results showed lymphoid vasculitis, and two mechanisms were considered to play a central role in the onset and progression of cardiovascular lesions in CAEBV: EBV-infected T or NK lymphocyte infiltration and injuries in the myocardium and vessel walls, and EBV-induced high levels of pro- and anti-inflammatory cytokines resulting in inflammatory responses [12, 14, 15]. As for the pathogenesis of CAE or CAA, the activation of matrix-degrading enzymes (especially matrix metalloproteinases [MMPs]) and enzymatic degradation of the extracellular matrix (ECM) of the media are considered to be the most critical molecular events, which ultimately lead to excessive expansive arterial remodeling [9, 16]. This process is mediated via several factors, including increased levels of inflammatory mediators [e.g., vascular endothelial growth factor (VEGF), adhesion molecules], and induction of nitric oxide (NO) and its metabolite, which could trigger MMP formation [9, 16]. These factors have also been shown to play an important role in the pathogenesis of CAA in Kawasaki disease (the most common cause of CAA in childhood), but their functions in CAEBV still need to be further elucidated [17–19]. It has been noted that CAA frequently occurs in association with more widespread vascular abnormalities, including aneurysms in the thoracic and abdominal aorta, as well as in the pulmonary and iliac arteries [20]. Our patient also suffered from segmental dilation of the abdominal aorta.

The reports of CAEBV-associated uveitis are rare, and the exact mechanisms are not fully understood. Infectious uveitis could arise from local infection, but is more commonly due to hematogenous spread of pathogens to the uvea [10]. The pathogenic antigens are presented to the leukocytes within the eye that are activated against infectious agents, and the release of chemokines could further attract leukocytes to the inflammation sites [21]. Therefore, uveitis occurs as collateral damage from immune responses and is the result of the breach of the blood-retinal barrier that occurs due to the inflammatory cascade [10, 21]. Wong et al. [22] described three CAEBV patients whose ocular involvement ranged from anterior uveitis to a severe panuveitis with cataract, vitritis, macular edema, and optic disc swelling. The onset age of three reported patients was between 15 years old and 30 years old, and they suffered from uveitis almost simultaneously with the CAEBV diagnosis or nearly 2 years after the diagnosis. Although their ocular lesions were relieved for a while by glucocorticoid and/or acyclovir therapies, the uveitis could relapse repeatedly [22]. Morishima et al. [36] reported a 7-year-old girl with CAEBV and associated uveitis who exhibited bilateral granulomatous iridocyclitis, mild vitritis, optic disk swelling, and left facial nerve palsy nearly 2 years after the diagnosis of CAEBV. Treatment with topical steroids, systemic interleukin-2, and splenectomy relieved the symptoms [23]. There have been no reports about the direct relationship between CAEBV and cataracts. However, cataract development is common among children with uveitis and is strongly related to the extent of inflammation recurrences [24]. Therefore, we believe the cataract of our patient may be secondary to her uveitis. She received a cataract operation after the HSCT, after which her vision recovered gradually.

In summary, this atypical CAEBV case with CAA and uveitis could help us recognize similar cases more easily, make the right diagnosis as early as possible, and deliver proper and timely treatment.

## Data Availability

All the data and materials used in this report are included in the manuscript.

## Abbreviations

allo-HSCT: Allogeneic HSCT
CAA: Coronary artery aneurysm
CAE: Coronary artery ectasia
CAEBV: Chronic active Epstein–Barr virus infection
CMV: Cytomegalovirus
EBERs: EBV-encoded small RNAs
ECM: Extracellular matrix
HHV-8: Human herpes virus 8
HIV: Human immunodeficiency virus
HLH: Hemophagocytic lymphohistiocytosis
HSCT: Hematopoietic stem cell transplantation
HSV: Herpes simplex virus
IM: Infectious mononucleosis
LPDs: Lymphoproliferative disorders
NO: Nitric oxide
PAH: Pulmonary arterial hypertension
PIDs: Primary immunodeficiency diseases
MMPs: Matrix metalloproteinases
VEGF: Vascular endothelial growth factor
VZV: Varicella zoster virus
WES: Whole-exome sequencing
